# A complex COVID-19 case with rheumatoid arthritis treated with tocilizumab

**DOI:** 10.1007/s10067-020-05234-w

**Published:** 2020-06-19

**Authors:** Shaozhe Cai, Wei Sun, Ming Li, Lingli Dong

**Affiliations:** 1 Department of Rheumatology and Immunology, Tongji Hospital, Huazhong University of Science and Technology, No. 1095 Jiefang Avenue, Wuhan, 430030 China; 2 Department of Stomatology, Union Hospital, Huazhong University of Science and Technology, No. 1277 Jiefang Avenue, Wuhan, 430022 Hubei China; 3 Department of Respiratory and Critical Care Medicine, Wuhan Pulmonary Hospital, No. 28 Baofeng Road, Wuhan, China

**Keywords:** Coronavirus disease 2019, Rheumatoid arthritis, Secondary opportunistic infection, Tocilizumab

## Abstract

Recurrences of COVID-19 were observed in a patient with long-term usage of hydroxychloroquine, leflunomide, and glucocorticoids due to her 30-year history of rheumatoid arthritis (RA). Tocilizumab was applied and intended to target both COVID-19 and RA. However, disease of this patient aggravated after usage of tocilizumab. After the discussion of a multiple disciplinary team (MDT) including rheumatologists, antimicrobial treatments were applied to target the potential opportunistic infections (*Pneumocystis jirovecii* and *Aspergillus fumigatus*), which were authenticated several days later via high throughput sequencing. As an important cytokine in immune responses, IL-6 can be a double-edged sword: interference in the IL-6-IL-6 receptor signaling may save patients from cytokine release storm (CRS), but can also weaken the anti-infectious immunity, particularly in rheumatic patients, who may have received a long-term treatment with immunosuppressive/modulatory agents. Thus, we suggest careful considerations before and close monitoring in the administration of tocilizumab in rheumatic patients with COVID-19. Besides tocilizumab, several disease-modifying antirheumatic drugs (DMARDs) can also be applied in the treatment of COVID-19. Therefore, we also reviewed and discussed the application of these DMARDs in COVID-19 condition.

**Electronic supplementary material:**

The online version of this article (10.1007/s10067-020-05234-w) contains supplementary material, which is available to authorized users.

Coronavirus disease 2019 (COVID-19) has progressed to a worldwide pandemic situation, and millions of people are suffering from this lethal disease. Many cytokines involved in the pathogenesis of rheumatic diseases (particularly rheumatoid arthritis, RA), such as IL-6, were elevated in COVID-19 [1–3]. Persistent and dramatic elevation of serum IL-6 level was associated with higher mortality in COVID-19 patients [4]. It seems that administration of tocilizumab can be a perfect solution to treat COVID-19 and RA at the same time. However, every coin has two sides. Application of tocilizumab may bring unsuspected effects. Here, we first reported the complex treatment process of a COVID-19 patient with RA history and illustrated the importance of recognizing the feasibility of tocilizumab application in the therapeutic process.

## Case presentation

The case we reported here was a 72-year-old female with a history of RA for nearly 30 years. She had taken leflunomide (LEF, 20 mg po qd) for 10 years and hydroxychloroquine (HCQ, 0.2 g po bid) for 1 year. Based on the clinical characteristics and therapeutic processes, this report was divided into two parts.

### Part 1 (Fig. 1)

**Fig. 1:**
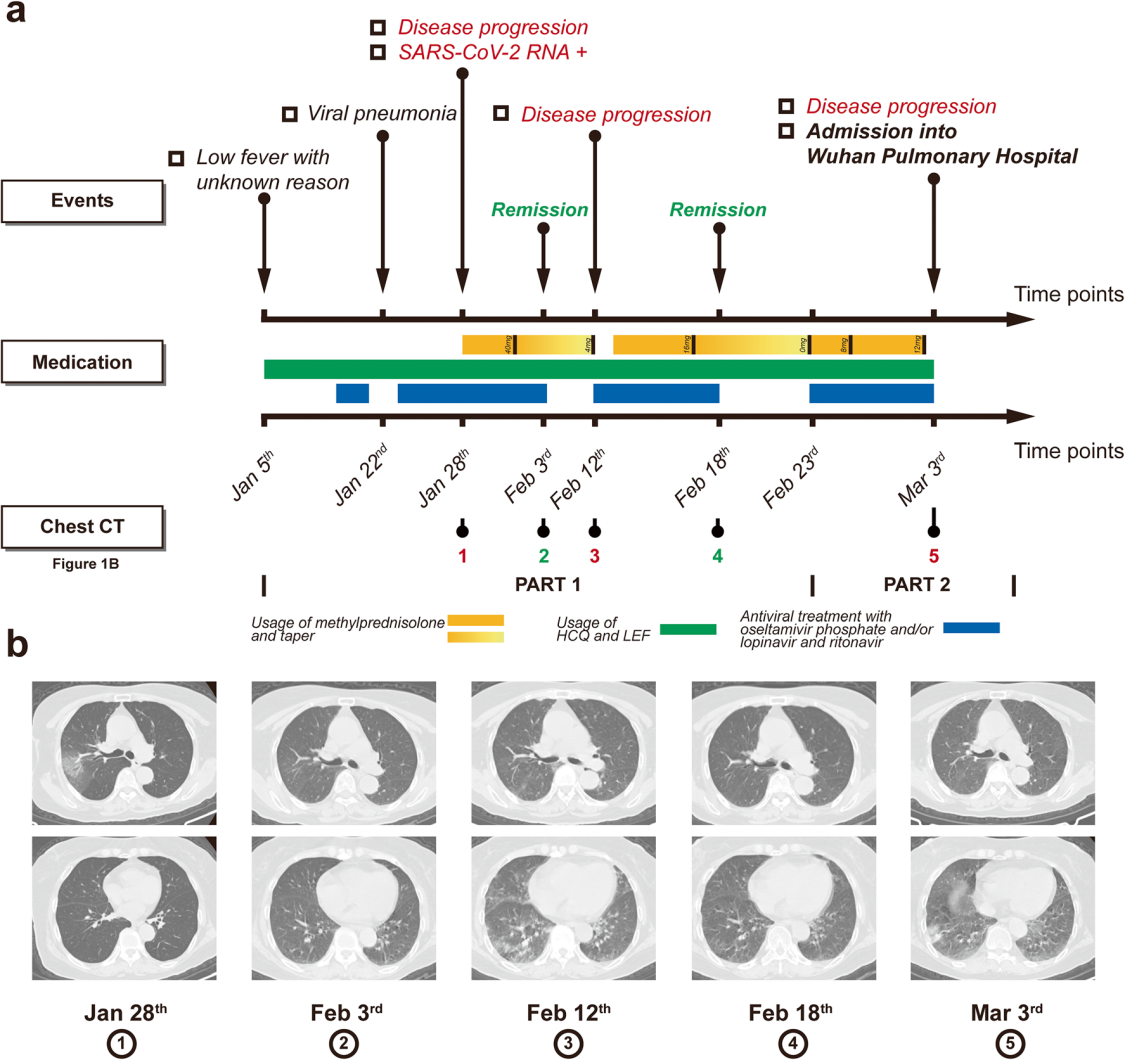
The 1st part of therapeutic processes of the case reported in this study. **a** Important events, medication, and radiologic features of this case before March 3, 2020 (Part 1). **b** Images of chest CT indicated in the corresponding panel of **a**

Fever of unknown reason (< 38 °C) emerged in this case on January 5, 2020, accompanied with cough, expectoration (white, but little), and mild shortness of breath. Chest CT showed pneumonia in the right upper lobe of her lungs. Positivity of severe acute respiratory syndrome coronavirus 2 (SARS-Cov-2) RNA was detected in her swab samples (January 28), and chest CT on the day before showed progression of the lesions in her right upper lobe (RUL) (Fig. 1a (b1)). Thus, a diagnosis of COVID-19 was made. After administration of antiviral agents (oseltamivir phosphate and lopinavir and ritonavir) and methylprednisolone (40 mg po qd) for 5 days, a significant relief of cough and breath shortness was observed. Chest CT on February 3 showed significant absorption lesions in her lungs (Fig. 1a (b2)). Dosage of glucocorticoids started to be tapered since January 31, and usage of antiviral agents was stopped on February 4. However, when usage of methylprednisolone was quickly tapered to 4 mg/day (February 11) within 11 days, her body temperature rebounded to 38.4 °C and ground glass opacities (GGOs) and patchy shadows appeared in both of her lungs (Fig. 1a (b3)). Antiviral treatment (lopinavir and ritonavir) restarted and dosage of methylprednisolone was elevated to 16 mg/day. Six days later, her body temperature returned to normal, and lesions in her lungs were absorbed totally (Fig. 1a (b4)). Administration of lopinavir and ritonavir was then stopped (February 18), and taper of methylprednisolone started. But fever emerged again after usage of methylprednisolone (10 days) was stopped (February 23). Then, another similar round of therapy was made. On March 2, she presented at the outpatient station with mild fever (37.7 °C), accompanied with chest tightness and shortness of breath. Due to the recurrence and progression of the disease, she was received in Wuhan pulmonary hospital on March 3rd (Figs. 1 (b5) and 3a).

### Part 2 (Figs. 2 and 3)

The laboratory indices showed a decreased lymphocyte count (0.34 * 10^9^/L) in peripheral blood and elevated levels of erythrocyte sedimentation rate (ESR, 39 mm/h) and C-reactive protein (CRP, 20.53 mg/L). The serum IL-6 level (6.77 pg/mL) was in normal range (< 7 pg/mL). Detection of anti-SARS-CoV-2 antibody showed negativity of IgM subtype, but strong positivity of IgG subtype. Four days later (March 7), her ESR elevated to 66 mm/h, and serum IL-6 level elevated to 115.4 pg/mL. Chest CT showed increased lesions in both of her lungs (Fig. 3b). Severe type of COVID-19 was considered and tocilizumab (400 mg iv drip), which was intended to target both COVID-19 and RA, was used in this case. Dosage of glucocorticoid use was also increased. Despite the escalation of treatment, the patient’s serum IL-6 level was still maintained on a high level, and her condition went down sharply on March 10 (Fig. 3c). On March 14, her serum IL-6 level elevated to 260.1 pg/mL, and chest CT showed even worse condition on both her upper lungs (Fig. 3d). Tocilizumab (400 mg iv drip) was then administrated for the second time. Unfortunately, this patient’s condition was going from bad to worse: on March 17, her dyspnea severed (oxygen flow was escalated to 40 to 50 L/min, and she could not even move on the bed), serum concentration of IL-6 surged to 2055 pg/mL, and obvious aggravation of lesions was observed on her upper lungs (Fig. 3e). This alerted the physicians, that the clinical manifestations of this case and the disease aggravation could not be explained, at least solely, by the potential infection of SARS-CoV-2. Therefore, a multiple disciplinary team (MDT) including rheumatologists was formed and discussed to find out the potential reasons leading to the current situation of this case. Infection of other pathogens due to her over suppressed immune systems (resulted from the administration of tocilizumab and/or glucocorticoids) was considered as the most possible reason. We started to detect the presence of potential pathogenic pathogens in this case immediately. Administration of LEF was stopped, and antiviral (ganciclovir sodium, 500 mg qd ivdrip), antibacterial (cefoperazone sodium sulbactam sodium, 3 g bid ivdrip), and antifungal (caspofungin acetate, 70 mg [for the first day]/50 mg qd ivdrip) agents were then administrated at the same time. Meanwhile, we also noticed that the serum ferritin level of this case was abnormally high (2442 μg/L), platelet count decreased continuously to 83 * 10^9^/L, and hypofibrinogenemia presented (1.9 g/L). All these indicated the tendency of secondary hemophagocytic lymphohistiocytosis (sHLH) characterized by a cytokine storm (which was authenticated by the elevated serum IL-6, IL-2R, IL-8, and TNF-α level detected several days later on March 21), hemophagocytosis, and further multi-organ damage. Thus, the administration of methylprednisolone was also escalated. The situation seemed to be turning: absorption of lesions was observed on chest CT on March 20 (Fig. 3f). On March 22, the high throughput sequencing analysis reported detection of *Pneumocystis jirovecii* and *Aspergillus fumigatus*. Chest CT on March 24 showed improvement (Fig. 3g), and levels of serum cytokines dropped significantly. In combination with antimicrobial treatment and application of blood products, this case was brought back from death: her chest CT on March 31 (Fig. 3h) and April 8 (Fig. 3i) both showed significant absorption of lesions on her lungs, and the oxygen flow was gradually reduced to 2 L/min: she could finish daily activity without too much effort. After the final detection of SARS-CoV-2 (RNA−, IgM−, IgG±) and CMV (DNA−), she was discharged on April 14. The detailed medication in this part was recorded in Supplementary Fig. 1.

**Fig. 2:**
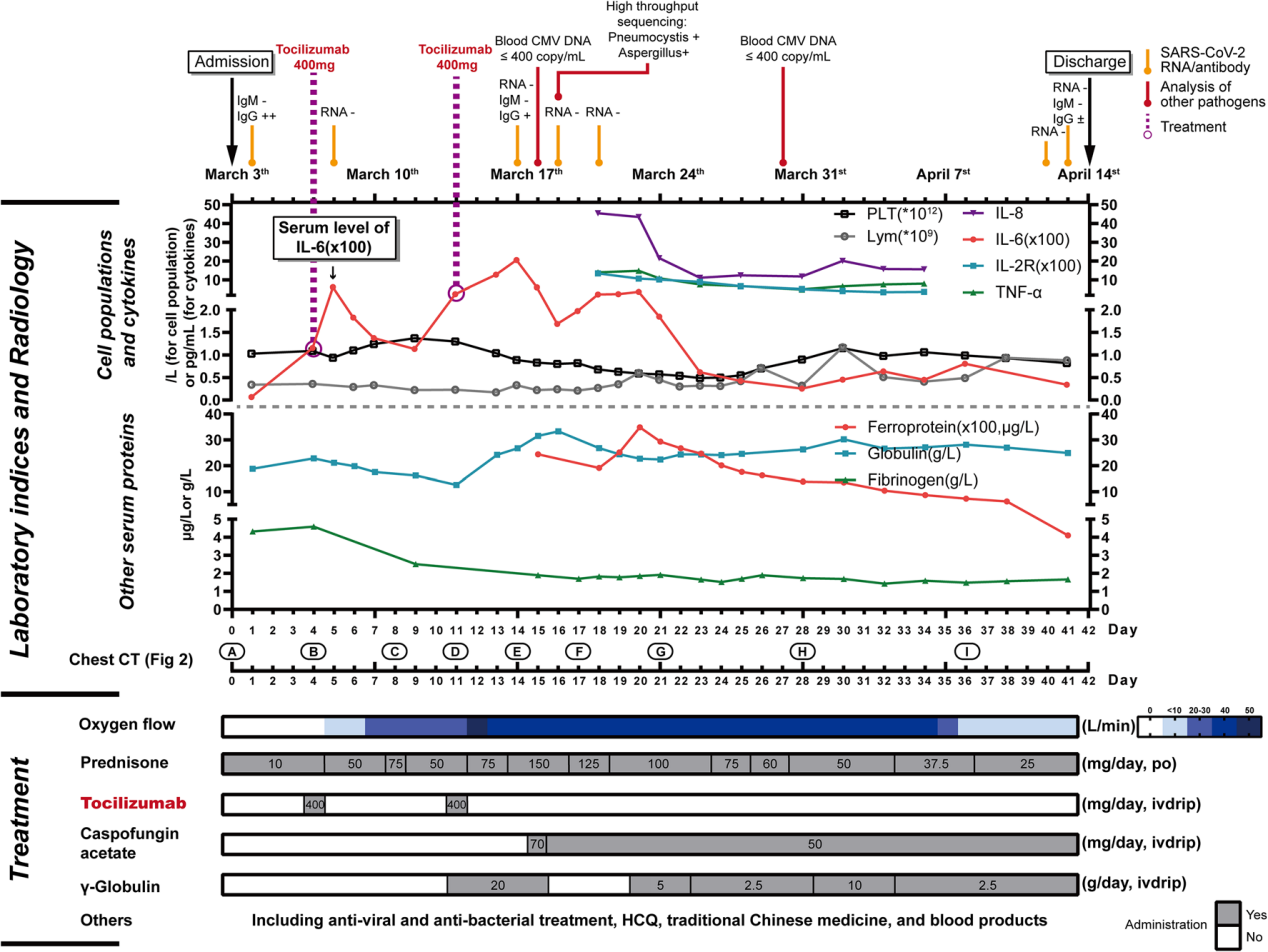
The 2nd part of therapeutic processes of the case reported in this study. Details of therapeutic process of this case after the hospitalization on March 3 due to disease recurrence and progression (Part 2). RUL right upper lobe, RLL right lower lobe, ESR erythrocyte sedimentation rate, CRP C-reactive protein, IL interleukin, TNF tumor necrosis factor, Lym lymphocyte, PLT platelet, SaO_2_ arterial oxygen saturation

**Fig. 3:**
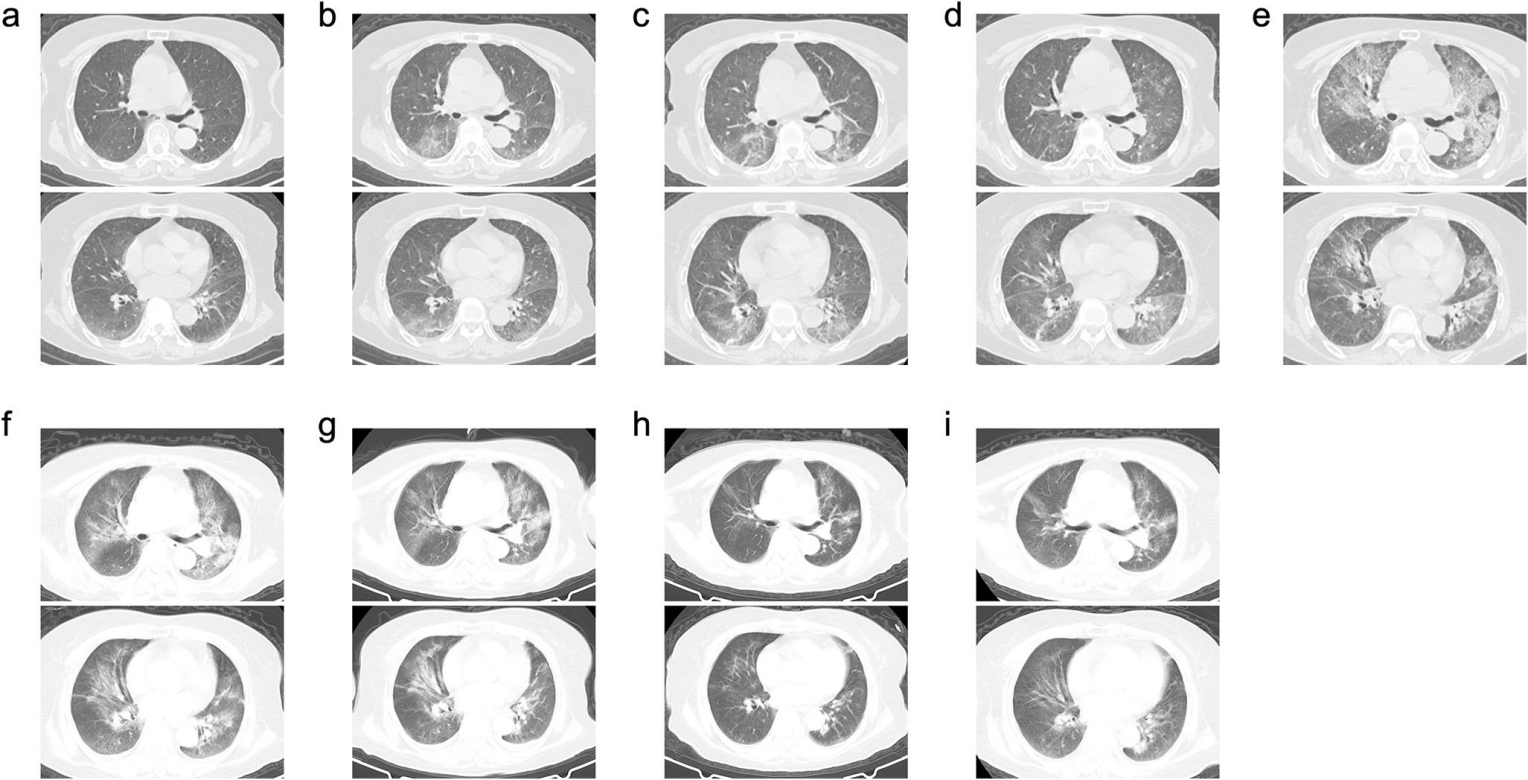
Radiology (Chest CT) of the case reported in this study during the hospitalization after March 3. The date of examination and the corresponding disease status at that time were recorded in Fig. 2

## Review and discussion

Up to the end of April 2020, more than 2.5 million people in the world had been infected with SARS-CoV-2 under the pandemic situation of COVID-19 [5]. As a large population with underlying dysregulated immune system, rheumatic patients infected with SARS-CoV-2 are not rare. How to improve the therapeutic efficacy for patients in this situation is an important task and challenges for physicians.

### Therapy with conventional synthetic disease-modifying antirheumatic drugs (cs-DMARDs) and glucocorticoids

HCQ and LEF are both important components of cs-DMARD in treating rheumatic diseases. Besides that, both of them showed antiviral properties, particularly HCQ, whose analogue chloroquine (CQ) is effective in inhibiting the infection and spread of SARS coronavirus (SARS-CoV) via interfering with terminal glycosylation of ACE2, which is also the entry receptor of SARS-CoV-2 [6, 7]. Some clinical investigations also showed efficacy of HCQ in treating mild to moderate COVID-19 patients [8, 9]. Thus, we applied these two agents in this case at the beginning of the treatment and intended to intervene with her COVID-19 and RA at the same time. However, with the deepening of research, side effects (e.g., cardiotoxicity) in chloroquine- or hydroxychloroquine-treated COVID-19 patients, especially when used at a high dose, were reported [10, 11]. There was also report showing no effects of HCQ in patients hospitalized for COVID-19 infection with oxygen requirement [12].

Glucocorticoids are another agent commonly seen in the treatment of both rheumatic diseases and COVID-19. The anti-inflammatory effects of glucocorticoids are always rapid and significant and are commonly used in the suppression of strong and harmful inflammatory process in pathophysiologic conditions. However, slow-down of the clearance of virus (including SARS-CoV-2) is always observed in the infected cases with systemic usage of glucocorticoids [13–15]. This might be the reason why the recurrence of COVID-19 happened in the case we reported here.

### COVID-19 and rheumatic diseases share similar pathogenic cytokines

At the level of pathogenic mechanisms, cytokines play important role in the progress of both COVID-19 and rheumatic diseases: Huang et al. revealed elevated serum level of many cytokines (including IL-1β, IL-7, IL-8, IL-10, GM-CSF, IFN-γ, TNF-α, etc.) in COVID-19 patients compared with healthy people, and these cytokines are also the pathogenic factors in many rheumatic diseases, including RA, systemic lupus erythematosus (SLE), and primary Sjögren’s syndrome (pSS) [2, 3, 16, 17]. Thus, targeting these potential pathogenic proinflammatory cytokines is logical and can be a good strategy to realize the win-win mode to treat both COVID-19 and the underlying rheumatic conditions. Among all these candidate target cytokines, IL-6 seems to be one of the best choices, particularly for COVID-19 patients with RA: from the aspects of either availability of the products or the current evidences supporting the benefits in treating both of the diseases [4, 18–21].

### Function of IL-6 and consideration needed in the administration of tocilizumab

IL-6 is an important cytokine in immune reactions, which can enhance the immune response (e.g., via promoting antibody production, differentiation of cytotoxic T cell or type 17 helper T cell, etc.), and always acts as a mediator notifying the occurrence of some emergent event elicited by either pathogen-associated molecular pattern (PAMPs) and damage-associated molecular patterns (DAMPs) [22]. Its elevation can be a sign of cytokine release syndrome (CRS), which is always observed in severe COVID-19 patients [1–3, 23]. However, every coin has two sides; it can also be a part of sHLH, which can be triggered by malignancy, infection (particularly viral infection), autoinflammation alone, or a combination of these factors [24]. Due to the high mortality of sHLH, early identification and the following aggressive treatment to suppress the overactivated immune system and the underlying triggers are required. In this case, after we observed the abnormal elevation of serum ferritin level, and decrease of platelet count in the context of infections and probable autoinflammatory status, we perceived the tendency of sHLH and applied decisively large-dosage glucocorticoids (methylprednisolone) and antimicrobial agents, which were finally authenticated as the key treatment saving this patient from death. It is interesting and worth mentioning that the tendency of sHLH was identified by a rheumatologist, who might have more chances to deal with a specific form of sHLH occurring in the context of autoimmunity, that is, macrophage activation syndrome (MAS) [24].

## Electronic supplementary material

ESM 1 (DOCX 269 kb).
