# Supplementary material for: A complex COVID-19 case with rheumatoid arthritis treated with tocilizumab
Source: Clin Rheumatol. 2020 Jun 19;39(9):2797–802. doi: 10.1007/s10067-020-05234-w (PMC7303585; doi:10.1007/s10067-020-05234-w)
Supplement: Supplementary file 1 — (DOCX 269 kb). [file 10067_2020_5234_MOESM1_ESM.docx]

**Supplementary data**


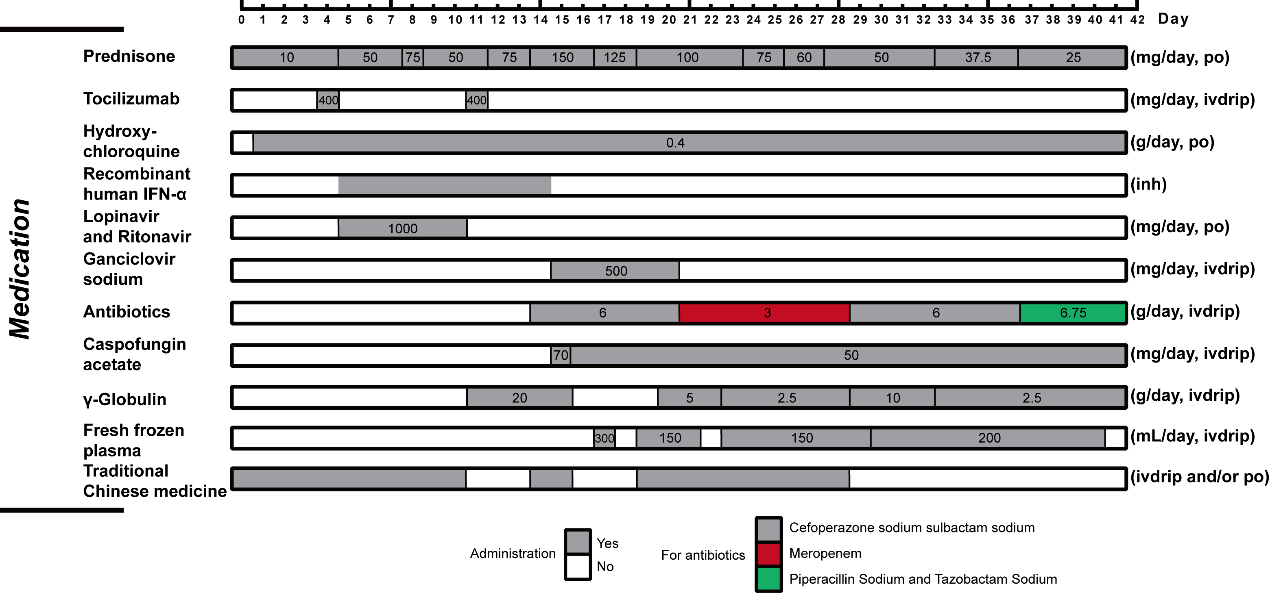


**Supplementary Fig. 1 Medication in detail of this patient in the 2^nd^ part of therapeutic process**
